# Purinergic Receptor Antagonists Inhibit Hemolysis Induced by *Clostridium perfringens* Alpha Toxin

**DOI:** 10.3390/pathogens13060454

**Published:** 2024-05-27

**Authors:** Zishuo Guo, Nan Yue, Ming Chen, Jiaxin Li, Ruomei Lv, Jing Wang, Tingting Liu, Jing Huang, Shan Gao, Yanwei Li, Bing Yuan, Jinglin Wang, Lin Kang, Bin Ji, Wenwen Xin

**Affiliations:** 1 State Key Laboratory of Pathogen and Biosecurity, Institute of Microbiology and Epidemiology, AMMS, Beijing 100071, China; g19961931739@163.com (Z.G.); yn18795116327@163.com (N.Y.); chenming3901@163.com (M.C.); lijiaxin@bmi.ac.cn (J.L.); lv_ruomei@163.com (R.L.); amms_wj@163.com (J.W.); liutingtingwushi@163.com (T.L.); huangjing@nieh.chinacdc.cn (J.H.); gaoshan@bmi.ac.cn (S.G.); liyanwei_00@163.com (Y.L.); yuanbing3008@126.com (B.Y.); wjlwjl0801@sina.com (J.W.); 2 Emergency Department, 96605 Army Hospital of the People’s Liberation Army, Jilin 134000, China; 3 Department of Disease Control, The Affiliated Wuxi Center for Disease Control and Prevention, Nanjing Medical University, Wuxi Center for Disease Control and Prevention, Wuxi 214101, China

**Keywords:** *Clostridium perfringens*, α-toxin, hemolysis, P2 receptor

## Abstract

*Clostridium perfringens* alpha toxin (CPA), which causes yellow lamb disease in sheep and gas gangrene and food poisoning in humans, is produced by all types of *C. perfringens* and is the major virulence determinant of *C. perfringens* type A. CPA induces hemolysis in many species, including humans, murines, sheep and rabbits, through its enzymatic activity, which dissolves the cell membrane. Recent studies have shown that some pore-forming toxins cause hemolysis, which is achieved by the activation of purinergic receptors (P2). However, the relationship between P2 receptors and non-pore-forming toxin hemolysis has not been investigated. In the present study, we examined the function of P2 receptors in CPA toxin hemolysis and found that CPA-induced hemolysis was dependent on P2 receptor activation, and this was also true for *Staphylococcus aureus* β-Hemolysin, another non-pore-forming toxin. Furthermore, we use selective P2 receptor antagonists to demonstrate that P2X1 and P2X7 play important roles in the hemolysis of human and murine erythrocytes. In addition, we found that redox metabolism was mainly involved in CPA-induced hemolysis using metabolomic analysis. We further demonstrate that CPA activates P2 receptors and then activates NADPH oxidase through the PI3K/Akt and MEK1/ERK1 pathways, followed by the production of active oxygen to induce hemolysis. These findings contribute to our understanding of the pathological effects of CPA, clarify the relationship between P2 activation and non-pore-forming toxin-induced hemolysis, and provide new insights into CPA-induced hemolysis.

## 1. Introduction

*Clostridium perfringens* is a Gram-positive spore-forming anaerobe that can cause many histotoxic and enterotoxic diseases in humans and animals [1]. *C. perfringens* produces more than 20 exotoxins and is categorized into seven toxin types, A to G, depending on which one or more of the major toxin genes (alpha, beta, epsilon, iota, CPE or Net B) it contains [2,3]. *C. perfringens* alpha toxin (CPA) is present in all strains of *C. perfringens* and is the key virulence of *C. perfringens* type A [4]. Listed as a provisional biotoxin on the International Biological Weapons Verification List, CPA has been widely studied [5].

CPA is a zinc metallophospholipase C and belongs to a group of related bacterial phospholipases C (PLCs) including *Clostridium bifermentans* PLC, *Bacillus cereus* phosphatidylcholine-specific phospholipase (PC-PLC) and *Clostridium novyi* γ-toxin, which contain essential zinc ions and are reversibly inactivated by EDTA or o-phenanthroline [4,6,7]. CPA is a 42.5 kDa single polypeptide with two domains. The ~250-amino-acid N-terminal domain is responsible for catalytic activity, although it is not immunoprotective. The ~120-amino-acid C-terminal domain mediates host-cell-binding activity in the presence of calcium and is immunoprotective [8]. Functionally, CPA has two modes of action. At high concentrations, it directly disrupts the eukaryotic cell membrane, leading to cell death. At low concentrations, it causes some degree of phospholipid hydrolysis, which further activates diacylglycerol (DAG)- and ceramide-mediated signal transduction pathways, leading to the massive production of several intracellular mediators [9]. Previous studies have shown that CPA is toxic to a variety of animal red blood cells (RBCs). However, little research has been conducted on the mechanism of rCPA-induced hemolysis, especially in human erythrocytes.

Purinergic (P2) receptors are widely distributed in the body and participate in the regulation of most physiological processes [10,11]. Extracellular nucleotides act on two distinct families of P2 receptors, the P2X receptor (ligand-gated ion channel-type receptor) and the P2Y receptor (G protein-coupled receptor), which are now relatively well understood [12]. P2X receptors are trimeric ATP-gated cation channels that can mediate the rapid flow of Na^+^, K^+^, and Ca^2+^, some members of which also play a role in the exchange of organic ions [13]. In mammals, there are seven P2X receptor subunits (P2X_1_–P2X_7_) [13]. P2Y receptors are G protein-coupled receptors and regulate various signaling events, including adenylyl cyclase, phospholipase C, and ion channel activation [10]. To date, eight P2Y receptors have been identified in mammals (P2Y_1_, P2Y_2_, P2Y_4_, P2Y_6_, and P2Y_11_–P2Y_14_).

Activation of the P2 receptor is involved in erythrocyte hemolysis caused by some bacterial toxins [14], such as α-Hemolysin (HlyA) from *Escherichia coli* [15] and α-toxin from *Staphylococcus aureus* [16]. Our previous work confirmed that *C. perfringens* epsilon toxin (ETX)-induced hemolysis is closely related to P2 receptors, especially the P2X_7_ and P2Y_13_ subtypes [14]. However, the relationship between P2 receptors and hemolysis of non-pore-forming toxins has not been investigated. We hypothesized that rCPA-induced hemolysis might require activation of P2 receptors and conducted experimental verification.

In this study, we investigated the function of P2 receptors in rCPA hemolysis using different subtypes of P2 receptor antagonists. Our finding is that rCPA-induced hemolysis is dependent on P2 receptor activation, which is a novel insight into CPA activity and mode of action. Furthermore, the roles of ATP and reactive oxygen species (ROS) in rCPA hemolysis were investigated and the mechanism of rCPA-induced hemolysis was preliminarily clarified.

## 2. Results

### 2.1. rCPA Causes Hemolysis in Human and Murine Erythrocytes

**Figure 1:**
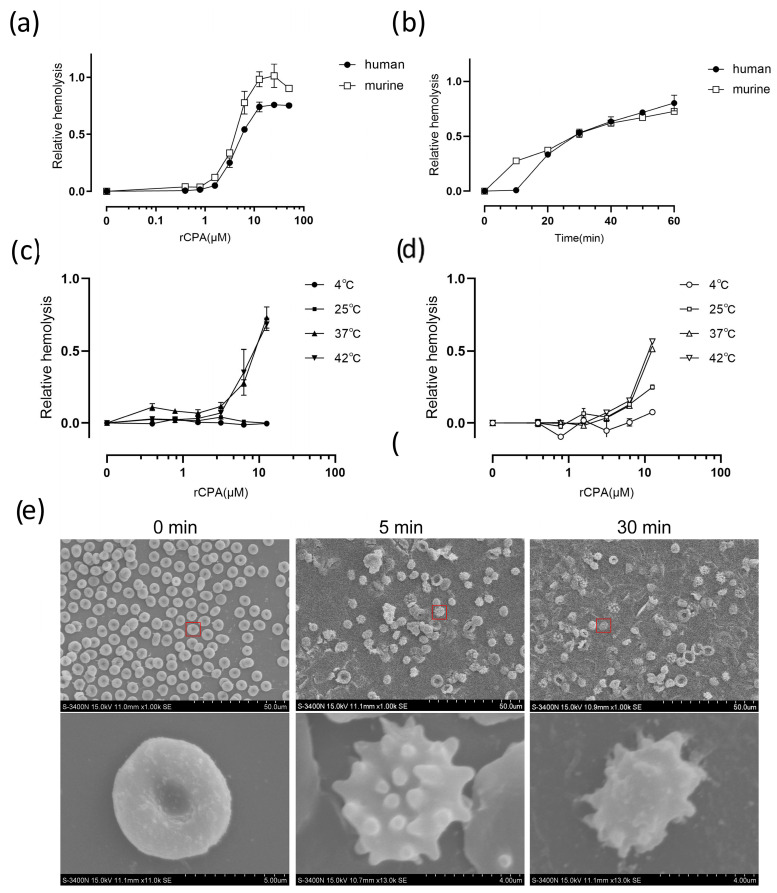
rCPA-induced hemolysis in human and murine erythrocytes. (**a**) Erythrocytes were incubated with rCPA (0–50 μM) for 60 min at 37 °C. A hemolysis value of 2% Triton-X 100 is defined as 1. (**b**) Extent of hemolysis with time. The erythrocytes were incubated with rCPA (50 μM) from 0 to 60 min at 37 °C. A hemolysis value of 2% Triton-X 100 is defined as 1. The hemolytic ability of rCPA was tested at various temperatures. (**c**) Human and (**d**) murine erythrocytes were incubated with rCPA (0–12 μM) for 60 min at 4 °C, 25 °C, 37 °C, and 42 °C. The hemolysis value of 2% Triton-X 100 is defined as 1. Values are the mean ± SD (*n* = 3). (**e**) Scanning electron microscopy images of human erythrocytes treated with rCPA. Human erythrocytes were incubated with rCPA (4.6 μM) for 0, 5, or 30 min at 37 °C and then attached to a coverslip.

Recombinant CPA (rCPA) with 6× His-tags at the C-terminal was expressed in the *E. coli* BL21 (DE3) strain and purified using a Ni^2+^-chelating affinity chromatography resin column. A high purity of rCPA protein was obtained (Figure S1). The hemolytic ability of rCPA was examined using erythrocytes from healthy volunteers and mice, at a final erythrocyte suspension concentration of 5%. The number of rCPA-lysed human and murine erythrocytes increased with the concentration of toxin (Figure 1a). rCPA-induced hemolysis was also time-dependent, with the level of hemolysis increasing with time (Figure 1b).

Temperature also played an important role in rCPA-induced hemolysis of human and murine erythrocytes (Figure 1c,d). Hemolysis was inhibited when cells were incubated at 4 °C or 25 °C. There was little difference in the degree of hemolysis at 42 °C compared to 37 °C, so 37 °C was chosen as the temperature used in subsequent experiments. The concentration of rCPA required for 50% hemolysis (HC_50_) after 60 min incubation at 37 °C was 4.6 μM. In all subsequent experiments, 4.6 μM of rCPA was therefore used.

To further survey the morphological effects of rCPA toxin on human erythrocytes, scanning electron microscopy was performed (Figure 1e). It can be seen from the figure that the normal red blood cells were smooth and round. When the rCPA toxin was in contact with the red blood cells for 5 min, it was observed that the red blood cells shrank and the volume decreased. At the time of 30 min, the red blood cell membranes shrank more seriously, some had broken, and hemolysis occurred.

### 2.2. rCPA-Induced Hemolysis Is Related to Activation of P2 Receptors

**Figure 2:**
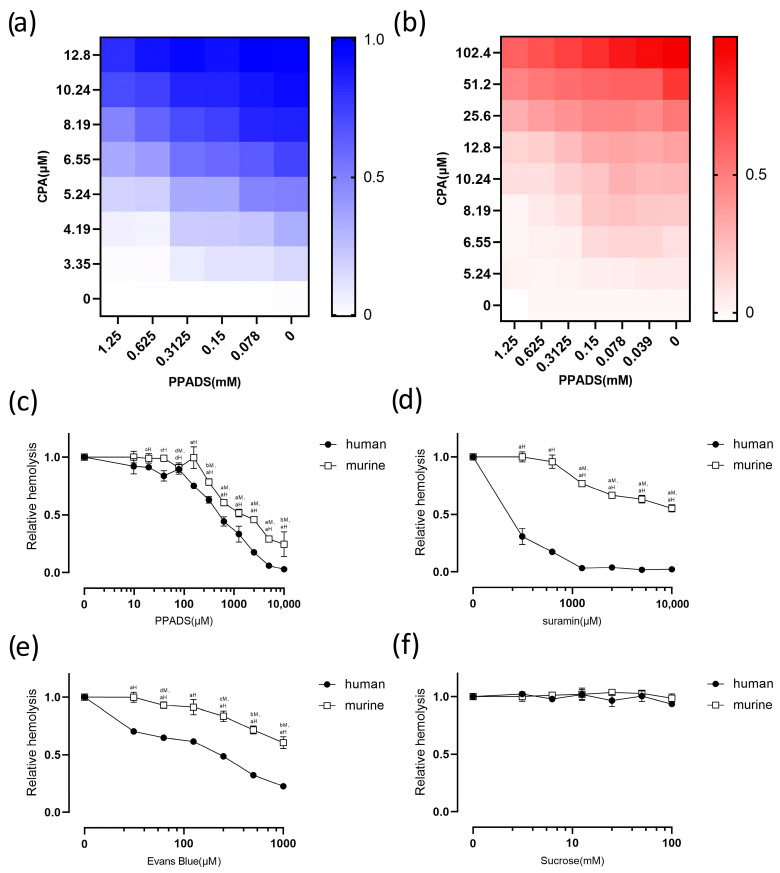
rCPA-induced hemolysis of human and murine erythrocytes is inhibited by non-selective purinergic receptor (P2) antagonists. Human (**a**) and mouse (**b**) erythrocyte hemolysis rates were derived from exposure to progressively increasing concentrations of rCPA and decreasing concentrations of PPADS for 1 h. (**c**) Effect of the non-selective P2 receptor antagonist PPADS on rCPA-induced lysis of erythrocytes. Hemolysis value corresponding to 0 μM PPADS and 4.6 μM rCPA was defined as 1. (**d**) The effect of suramin on rCPA-induced hemolysis of erythrocytes. Hemolysis value corresponding to 0 mM suramin and 4.6 μM rCPA was defined as 1. (**e**) The effect of Evans Blue on rCPA-induced hemolysis of erythrocytes. The hemolysis value corresponding to 0 μM Evans Blue and 4.6 μM rCPA was defined as 1. (**f**) The effect of increasing concentrations of sucrose on the hemolysis of human and murine erythrocytes. Values are the mean ± SD (*n* = 3). The hemolysis value corresponding to 0 mM sucrose and 4.6 μM rCPA was defined as 1. The statistical differences between each concentration of the inhibitor group and normal hemolysis group without inhibitor are indicated by letters a, b, c, and d, respectively, where “a” = *p* < 0.0001, “b” = *p* < 0.001, “c” = *p* < 0.01, “d” = *p* < 0.05, “H” denotes human, and “M” denotes mouse.

P2 receptor activation is reportedly involved in the pore-forming toxin-induced hemolysis of erythrocytes, such as the hemolysis induced by *E. coli* HlyA, ETX, and *S. aureus* α-toxin [14,15,16]. Consistent with this, we found that non-pore-forming rCPA-induced or Hlb-induced (*Staphylococcus aureus* β-Hemolysin) hemolysis was significantly inhibited by the non-selective P2 receptor antagonist PPADS, and this inhibition was concentration-dependent (Figure 2a–c) (Figures S2 and S3). The non-selective P2 receptor antagonist suramin also significantly decreased rCPA-induced hemolysis in a concentration-dependent manner (Figure 2d). The same phenomenon occurred in the non-selective P2X receptor antagonist Evans Blue (Figure 2e).

To eliminate the effects of osmolality changes, sucrose was used to change the extracellular osmotic pressure. Sucrose did not change the level of hemolysis, even at a concentration of 100 mM (Figure 2f). Considering that the concentrations of antagonists used in this study did not exceed 10 mM, the effect could not be the result of increased osmolality.

### 2.3. P2X_1_ and P2X_7_ Receptor Are Involved in rCPA-Induced Hemolysis

P2 receptors, including P2X_1_, P2X_2_, P2X_7_, P2Y_1_, P2Y_2_, and P2Y_13_, are expressed in various types of erythrocytes [17]. As there are no specific antagonists for P2X_2_ and P2Y_2_ receptors and low predicted expression levels of P2Y_2_ receptors on erythrocytes [17,18], the effects of P2X_2_ and P2Y_2_ receptors were not examined. In this study, we used different antagonists to test whether P2X_1_, P2X_7_, P2Y_1_, and P2Y_13_ were responsible for rCPA-induced hemolysis (Table 1).

Evans Blue, a non-selective P2X receptor inhibitor, significantly reduced rCPA-induced hemolysis, suggesting that P2X receptors are involved in this process (Figure 2e). Due to the presence of P2X receptors on the RBCs, we regarded P2X_1_ and P2X_7_ as the most likely mediators of rCPA-induced hemolysis [15,17]. The P2X_1_ and P2X_7_ receptors have also been reported to be involved in multiple-toxin-induced hemolysis of erythrocytes [15,16,19].

**Figure 3:**
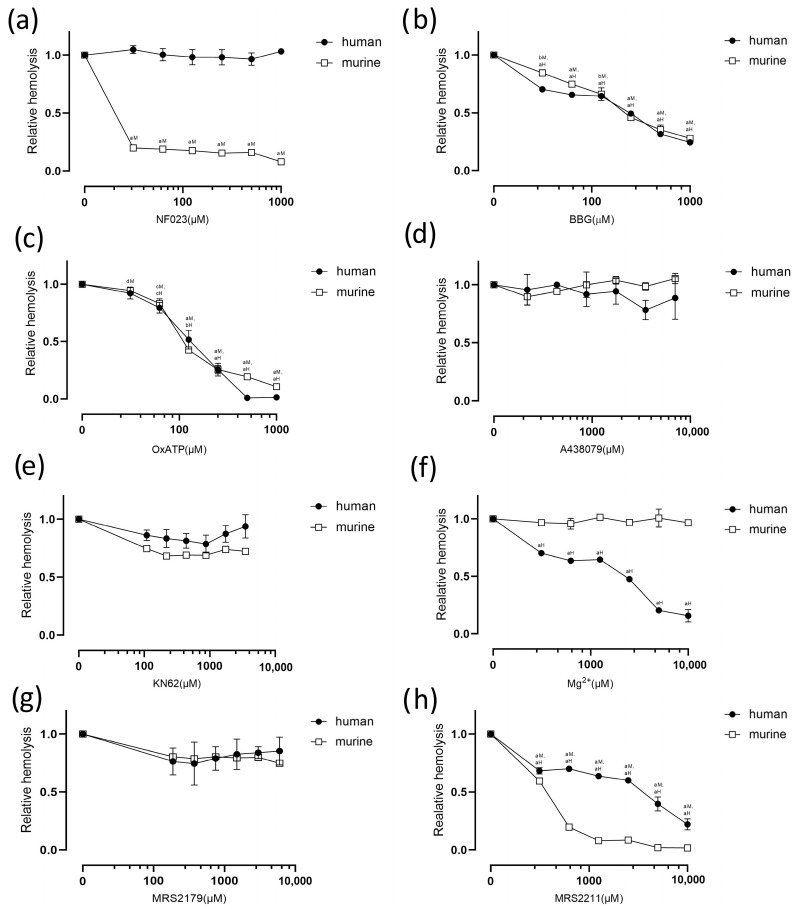
rCPA-induced hemolysis is inhibited by different antagonists. (**a**) Hemolysis of murine erythrocytes was inhibited by NF023. Hemolysis induced by rCPA was reduced by increasing concentrations of (**b**) BBG and (**c**) OxATP. rCPA-induced hemolysis was unaffected by difference even by high concentrations of (**d**) A438079 and (**e**) KN-62. (**f**) Mg^2+^ inhibited the hemolysis of human erythrocytes. Compared with (**g**) P2Y1 receptor inhibitor MRS2179, (**h**) P2Y_13_ receptor inhibitor MRS2211 significantly inhibited the hemolysis. Values are the mean ± SD (*n* = 3). The hemolysis value corresponding to 0 μM of each antagonist and 4.6 μM rCPA was defined as 1. The statistical differences between each concentration of the inhibitor group and normal hemolysis group without inhibitor are indicated by letters a, b, c, and d, respectively, where “a” denotes *p* < 0.0001, “b” denotes *p <* 0.001, “c” denotes *p* < 0.01, “d” denotes *p* < 0.05, H denotes human red blood cells, and M denotes mouse red blood cells.

The P2X_1_-selective antagonist, NF023, was used to study the effect on rCPA-induced hemolysis [20,21]. NF023 did not affect the rCPA-induced hemolysis of human erythrocytes. However, it significantly inhibited the hemolysis of erythrocytes from mice (Figure 3a).

P2X_1_- and P2X_7_-selective antagonists, Brilliant Blue G (BBG) and ATP-2′, 3′-dialdehyde (OxATP), significantly reduced hemolysis in a concentration-dependent manner (Figure 3b,c). To our surprise, by contrast to BBG and OxATP, the P2X_7_-selective antagonists A438079 and KN-62 only had a slight effect on the hemolysis of human and murine erythrocytes (Figure 3d,e).

However, we cannot exclude the possibility that the P2X_7_ receptor is partially involved in rCPA-induced hemolysis in human and murine erythrocytes because of the similar inhibitor profiles for P2X_1_ and P2X_7_ [17,22]. To address this, we used Mg^2+^ as a highly selective antagonist of the P2X_7_ receptor [22]. Mg^2+^ did not inhibit CPA enzymatic activity (Figure S4) but significantly inhibited rCPA-induced hemolysis in a concentration-dependent manner (Figure 3f), indicating that the P2X_7_ receptor is involved in the hemolysis of human erythrocytes.

We used the same methods to verify the effect of P2Y receptors. The selective P2Y_1_ receptor antagonist MRS2179 did not inhibit hemolysis (Figure 3g). The selective P2Y_13_ receptor antagonist MRS2211 significantly decreased hemolysis, indicating that the P2Y_13_ receptor is involved in rCPA-induced hemolysis (Figure 3h). Interestingly, ADP, another antagonist of the P2Y_13_ receptor, did not inhibit hemolysis (Figure S5). All results are summarized in Table 1.

### 2.4. rCPA-Induced Activation of P2 Receptors Is Not Mediated by ATP

**Figure 4:**
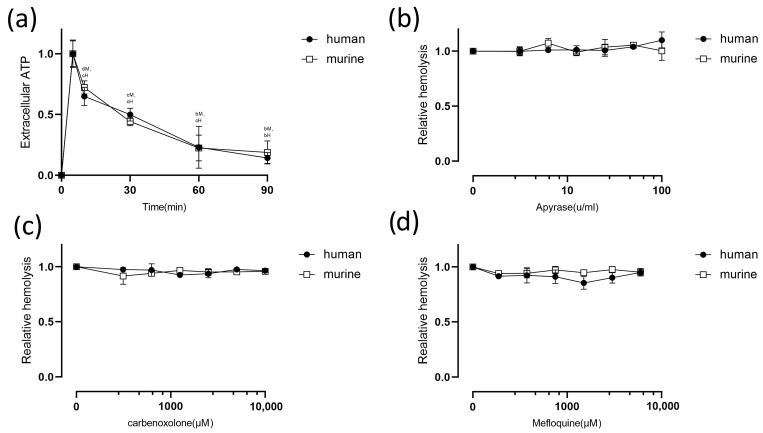
Relationship between rCPA-induced hemolysis of human and murine erythrocytes and extracellular ATP. (**a**) Adding rCPA (4.6 μM) to erythrocytes caused a rapid increase in extracellular ATP content from 0 to 5 min, and then a time-dependent decrease. The ATP value corresponding to 5 min is defined as 1. (**b**) The relative hemolysis of human erythrocytes incubated with increasing concentrations of the ATP-scavenging enzyme apyrase. Erythrocytes were incubated with rCPA (4.6 μM) and (**c**) carbenoxolone or (**d**) mefloquine for 60 min at 37 °C. Values are the mean ± SD (*n* = 3). The hemolysis value corresponding to 0 μM of each antagonist and 4.6 μM rCPA was defined as 1. The statistical differences between each concentration of the inhibitor group and normal hemolysis group without inhibitor are indicated by letters b, c, and d, respectively, where “b” denotes *p* < 0.001, “c” denotes *p* < 0.01, “d” denotes *p* < 0.05, H denotes human red blood cells, and M denotes mouse red blood cells.

Recent findings indicate that *E. coli* HlyA and *Aggregatibacter actinomycetemcomitans* leukotoxin A induce ATP release from RBCs by forming toxin pores, which then act on P2X_1_ and P2X_7_ to mediate hemolysis [15,19,23]. To assess this possibility in CPA, we tested the change in ATP at different reaction time points. A large amount of ATP was released when rCPA was added to the hemolysis system from 0 to 5 min (Figure 4a). The ATP-scavenging enzyme apyrase, however, had little effect on rCPA-induced hemolysis (Figure 4b). This indicates that rCPA-induced activation of P2 receptors is not mediated by ATP.

The P2X_7_ receptor has been reported to interact with the channel protein pannexin 1 [24], and this complex is involved in releasing ATP and Ca^2+^ [24,25,26,27]. To assess the effect of pannexin 1 on rCPA-induced hemolysis, carbenoxolone and mefloquine were used as antagonists of pannexin 1 (Figure 4c,d) [28,29]. The results showed that these two antagonists had almost no significant inhibitory effect on hemolysis, proving that pannexin 1 is not involved in rCPA-induced hemolysis.

### 2.5. Metabonomic Analysis of rCPA-Induced Hemolysis

**Figure 5:**
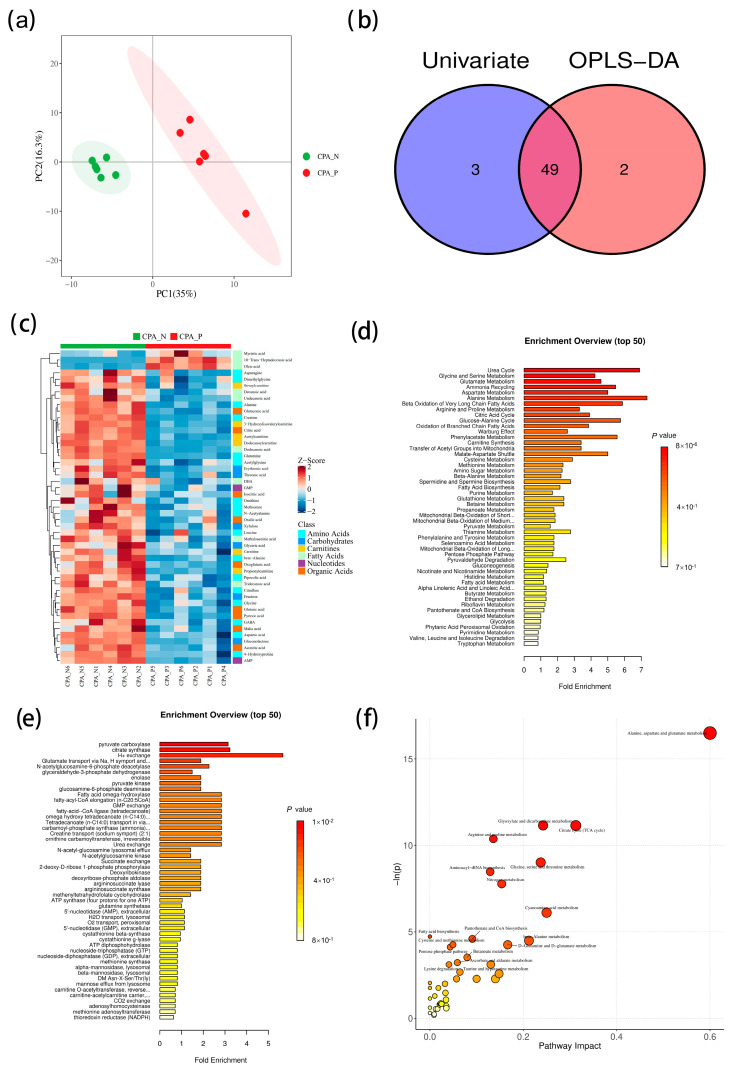
Metabolomic analysis of rCPA-induced hemolysis. (**a**) PCA score plot. (**b**) Venn Plot of differential metabolites. (**c**) Heat map of potential biomarkers. (**d**) Pathway enrichment analysis bar plot using pathway-associated metabolite sets (SMPDBs). (**e**) Pathway enrichment analysis bar plot using pathway-associated metabolite sets (predicted metabolite sets). (**f**) Pathway analysis bubble plot by hsa set.

The process of intracellular signal transmission is always accompanied by changes in enzymes and metabolic substrates. In order to further explore the cause of hemolysis caused by P2 receptor activation, the substrate changes during rCPA-induced human erythrocyte hemolysis were analyzed by metabolomics experiments. RBCs treated with rCPA for 10 min were immobilized and analyzed using targeted metabolomics. The intra-group and inter-group sample differences and possible outliers were analyzed using principal component analysis (PCA). In Figure 5a, each point represents a sample, the ellipse represents the 95% confidence interval, the abscissa (PC1) represents the first principal component, the ordinate (PC2) represents the second principal component, and the percentage in brackets represents the interpretation rate of the principal component. The plot shows that all samples are within the 95% confidence interval, and there is no overlap between the rCPA experimental group and the negative control group (RBCs not treated with rCPA). Thus, the difference in metabolites between the two groups was obvious and statistically significant.

Using the intersection of differential metabolites from univariate statistics and multivariate statistics, (*p* < 0.05 and |log2FC| > 0 in univariate statistics, variable importance in projection (VIP) >1 in multivariate statistics), we found 49 potential biomarkers with possible biological significance (Figure 5b). Heat maps of the 49 different metabolites were drawn (Figure 5c), among which three biomarkers were significantly upregulated, and the rest were downregulated.

Pathway-associated metabolite sets (SMPDBs) and predicted metabolite sets were used to conduct pathway enrichment analysis for the 49 metabolites (Figure 5d,e). We also used the Homo sapiens (human) set for pathway analysis; the bubble diagram of pathway analysis is shown in Figure 5f. The results suggest that the alanine, aspartic, and glutamate pathways are most affected by CPA. These results suggest that the redox metabolism is likely involved in rCPA-induced hemolysis, based on the three amino acids (alanine, aspartate, and glutamate) most involved in redox metabolism.

### 2.6. rCPA-Induced Hemolysis Leads to the Elevation of Reactive Oxygen Species

**Figure 6:**
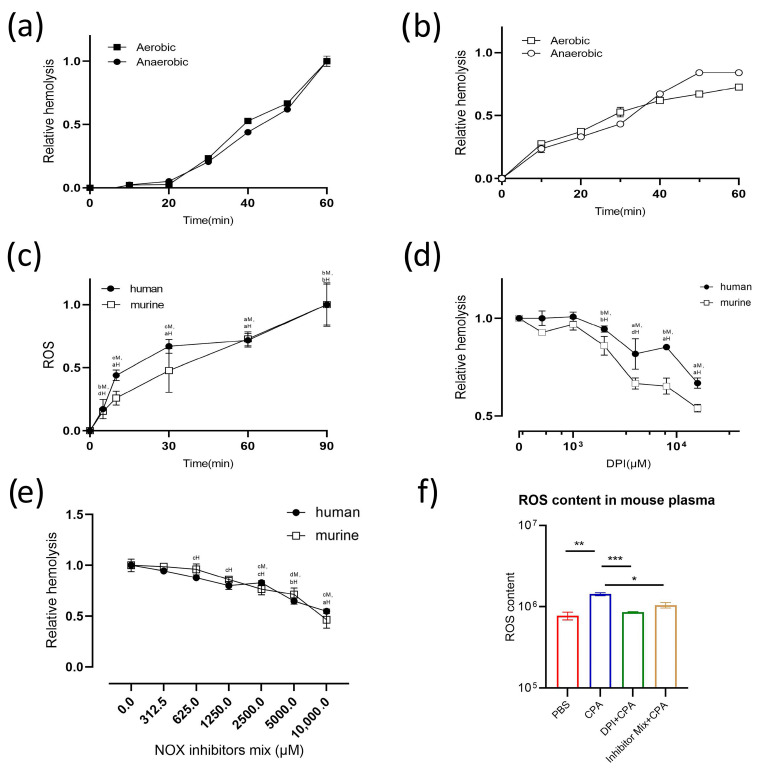
The effect of oxygen on rCPA-induced hemolysis. (**a**) Extracellular O_2_ has no effect on the rCPA-induced hemolysis of human and (**b**) murine erythrocytes. The rCPA concentration used was 4.6 μM. (**c**) Detection of extracellular ROS at different times of rCPA-induced hemolysis in human and murine erythrocytes. The rCPA concentration used was 4.6 μM. (**d**) DPI, an inhibitor of NADPH oxidase, can significantly inhibit the rCPA-induced hemolysis of human and murine erythrocytes. The hemolysis value corresponding to 0 μM of DPI and 4.6 μM rCPA was defined as 1. (**e**) Hemolysis of murine and human erythrocytes was inhibited by NOX inhibitors. Values are the mean ± SD (*n* = 3). The hemolysis value corresponding to 0 μM of inhibitor and 4.6 μM rCPA was defined as 1. The statistical differences between each concentration of the inhibitor group and normal hemolysis group without inhibitor are indicated by letters a, b, c, and d, respectively, where “a” denotes *p* < 0.0001, “b” denotes *p* < 0.001, “c” denotes *p* < 0.01, “d” denotes *p* < 0.05, H denotes human red blood cells, and M denotes mouse red blood cells. (**f**) NADPH oxidase inhibitors caused a decrease in ROS content in the plasma of mice. Among them, PBS was used as a negative control and rCPA as a positive control. The ROS content in the plasma of mice treated with rCPA (590 μg/kg) for 1 h was significantly increased. The ROS content in the plasma of mice pretreated with DPI (2 mg/kg/d, treatment for 3 days) and NOX inhibitor mix (20 mg/kg/d, treatment for 3 days) was significantly lower than that of the rCPA group. Values are means ± standard deviation. * *p <* 0.05, ** *p* < 0.01, *** *p* < 0.001 compared with CPA-treated positive controls.

According to clues achieved from metabonomic analysis, we explored the role of extracellular O_2_ and ROS in rCPA-induced hemolysis (Figure 6). Extracellular O_2_ did not play a part in the rCPA-induced hemolysis of human or murine erythrocytes (Figure 6a,b) and ROS production gradually increased as the degree of hemolysis increased (Figure 6c). This supports the hypothesis that redox metabolism is involved in rCPA-induced hemolysis. We further explored how ROS were generated. DPI (diphenyleneiodonium) was used to explore the role of NADPH oxidase (NOX) in rCPA-induced hemolysis (Figure 6d), as the production of ROS is closely related to NOX activation in erythrocytes [30,31,32,33]. We found that DPI significantly inhibited rCPA-induced hemolysis and the inhibitory effect was positively correlated with the inhibitor concentration. In addition, we found that several inhibitors of NOX can inhibit different isoforms of NOX separately (Table 2), so we mixed these four different inhibitors to explore their effects on hemolysis. The results showed that the mixture of these inhibitors could significantly inhibit rCPA-induced hemolysis (Figure 6e). We performed in vivo experiments in mice; mouse plasma ROS levels were measured after treatment with rCPA and inhibitor. The results showed that rCPA significantly increased the level of ROS in the plasma of mice, while the level of ROS in the plasma of mice that were pretreated with NADPH oxidase inhibitor significantly decreased after rCPA injection (Figure 6f). These results suggest that ROS may play a role in rCPA-induced hemolysis.

### 2.7. Signal Pathways Involved in P2 Receptor Activation and Reactive Oxygen Species Production

**Figure 7:**
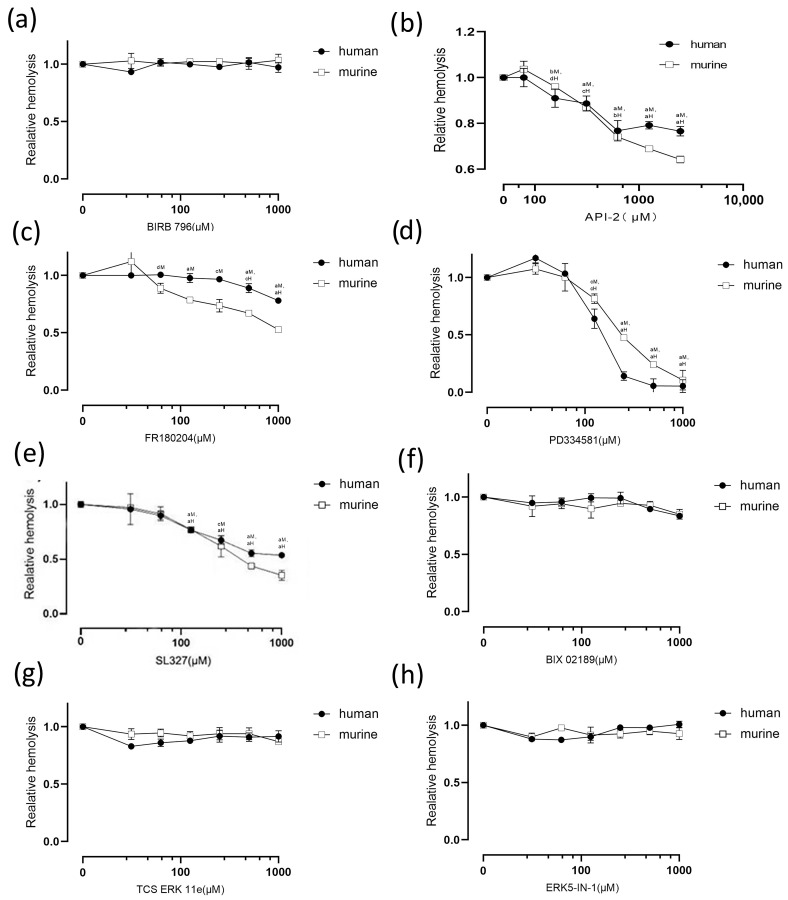
Effects of different pathway inhibitors on the rCPA-induced hemolysis of human and murine erythrocytes. (**a**) The degree of hemolysis did not change with increasing concentrations of the p38 MAPK inhibitor BIRB 796, implying no involvement in rCPA-induced hemolysis, in contrast to (**b**) the Akt inhibitor API-2, which can significantly inhibit rCPA-induced hemolysis. Regarding the MEK/ERK pathway, (**c**) the ERK inhibitor FR180204 slightly inhibits rCPA-induced hemolysis, (**d**) the MEK1 inhibitor PD334581 and (**e**) MEK1/2 inhibitor SL327 significantly inhibit rCPA-induced hemolysis, in contrast to (**f**) the MEK5/ERK5 inhibitor BIX02189, (**g**) ERK2 inhibitor TCS ERK 11e, and (**h**) ERK5 inhibitor ERK5-IN-1. Values are the mean ± SD (*n* = 3). The hemolysis value corresponding to 0 μM of each antagonist and 4.6 μM rCPA was defined as 1. The statistical differences between each concentration of the inhibitor group and normal hemolysis group without inhibitor are indicated by letters a, b, c, and d, respectively, where “a” denotes *p* < 0.0001, “b” denotes *p* < 0.001, “c” denotes *p* < 0.01, “d” denotes *p* < 0.05, H denotes human red blood cells, and M denotes mouse red blood cells.

Studies suggest that NADPH oxidase is activated in three ways: via the p38-MAPK, cx43, and MEK/ERK pathways [30,34,35]. Moreover, it has been shown that the activation of MEK1 is required for rCPA to induce ROS production and exert cytotoxic effects [36]. We used a p38-MAPK pathway inhibitor (BIRB 796) to explore the role of p38-MAPK in rCPA-induced hemolysis, and found that p38-MAPK does not appear to be involved in hemolysis (Figure 7a). The activation of cx43 is related to the phosphorylation of AKT, and thus, we applied an AKT inhibitor, API-2, to check the role of this pathway. API-2 significantly inhibited rCPA-induced hemolysis, indicating that rCPA-induced hemolysis may be mediated by the AKT pathway (Figure 7b). We studied the MEK/ERK pathway with inhibitors such as TCS ERK 11e, FR 180204, ERK5-IN-1, PD334581, SL327, and BIX 02189 (Figure 7c–h). The ERK non-selective inhibitor FR180204 inhibited rCPA-induced hemolysis, and the MEK1 inhibitor PD334581 and MEK1/2 inhibitor SL327 significantly inhibited rCPA-induced hemolysis, while the MEK5/ERK5 inhibitor BIX02189, ERK2 inhibitor TCS ERK 11e, and ERK5 inhibitor ERK5-IN-1 did not, indicating that the MEK1/ERK1 pathway but not the MEK5/ERK5 or MEK2/ERK2 pathways are mainly involved in the process of rCPA-induced hemolysis.

## 3. Discussion

The alpha toxin produced by *Clostridium perfringens* is an important virulence factor, causing food poisoning and gas gangrene in humans. As such, it is considered a potential bioterrorism threat. However, the pathogenesis of CPA has not yet been fully elucidated [37]. Over the past few decades, the biological activity of CPA has been tested and examined, including necrotic, lethal, phospholipase C, and sphingomyelinase activities [38]. However, the specific mechanism of rCPA-induced hemolysis remains unclear. This study reports on a new mechanism of hemolysis induced by rCPA that differs from that previously seen.

Hemolysis induced by pore-forming toxins such as HlyA and ETX requires the activation of P2 receptors [14,16]. Surprisingly, we found that P2 receptors are also involved in non-pore-forming rCPA-induced hemolysis. We then tested a series of antagonists to find out which P2 receptors were involved in rCPA-induced hemolysis using erythrocytes from two origins, human and mouse. We found that P2X_7_ and P2Y_13_ in human erythrocytes and P2X_1_ and P2Y_13_ in murine erythrocytes are key P2 receptors involved in rCPA-induced hemolysis. The P2Y_1_ receptor does not play a role in the hemolysis of rCPA toxin [39,40]. Functional P2Y_13_ is present on RBCs, where it is a G protein-coupled receptor that negatively regulates ATP release from the cell [18]. The selective P2Y_13_ receptor antagonist MRS2211 inhibited rCPA-induced hemolysis [15,16]. This finding contradicts our results with ADP (Figure S5), which should stimulate the ADP-sensitive P2Y_13_ receptor but has no effect on rCPA-induced hemolysis [15]. Thus, we conclude that P2Y_13_ was not involved in rCPA-induced hemolysis. Taken together, these data support the hypothesis that both the P2X_1_ and P2X_7_ receptors are relevant for rCPA-induced hemolysis. Unlike P2Y_13_ receptors, P2X_1_ and P2X_7_ receptors are ionic receptors. The P2X_1_ and P2X_7_ receptors are known to undergo a transition to a greater-permeability state, which eventually leads to lysis in certain cells [24]. P2X_1_ and P2X_7_ receptors are also reported to participate in HlyA-induced hemolysis [16] and ETX-induced hemolysis [14]. These findings indicate that P2X_1_ and P2X_7_ receptors are commonly involved in responses to both pore-forming and non-pore-forming toxins.

We hypothesize that a common signaling pathway between pore-forming and non-pore-forming toxin-induced hemolysis exists, in which P2 receptors play a key role. We tested another non-pore-forming toxin, *Staphylococcus aureus* β-Hemolysin (Hlb) (Figure S2), and found that P2 receptors were also involved in Hlb-induced hemolysis (Figure S3), indicating that P2 receptors are commonly involved in non-pore-forming hemolysin-induced hemolysis. Our results support the hypothesis that P2 receptors play a key role in hemolysis caused by both pore-forming and non-pore-forming toxins.

Recent studies have reported that ATP released from toxin-forming pores, such as HlyA from *E. coli* and leukotoxin A from *A. actinomycetemcomitans*, mediate hemolysis by acting on P2X_1_ and P2X_7_ receptors [15,17,19]. However, we found that the ATP scavenger apyrase cannot inhibit rCPA-induced hemolysis, which suggests ATP is not involved in the activation of P2 receptors. Pannexin 1 is also one of the pathways for ATP release, so we wondered if there was some previous association between pannexin 1 and P2 receptors [41,42]. The increases in extracellular ATP may lead to the opening of pannexin 1 through P2 receptors, which in turn causes an outflow of ATP, activating the P2 receptors. We thus tested the function of pannexin 1 in hemolysis but found that pannexin 1 did not play a role in rCPA-induced hemolysis. Therefore, the mode of ATP production during rCPA-induced erythrocyte hemolysis remains to be further explored.

**Figure 8:**
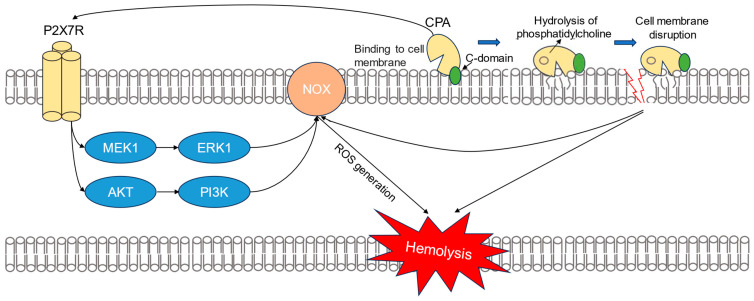
Inferred model for rCPA-induced hemolysis. First, rCPA binds to the membrane of human erythrocytes. Then, P2 receptors are activated by CPA. Next, the activation of P2 receptors leads to the activation of the PI3K/Akt and MEK1/ERK1 pathways, which leads to the production of ROS. This eventually leads to apoptosis of RBCs. In addition, the binding of CPA to the cell membrane leads to the hydrolysis of phosphatidylcholine in liposomes, which is also likely to be one of the causes of RBC hemolysis.

To further study rCPA-induced hemolysis, we conducted metabonomics experiments. Three kinds of amino acids (alanine, aspartate, and glutamate) were most involved in rCPA-induced hemolysis. Alanine, aspartate, and glutamate metabolisms, as important carbon metabolism pathways, are involved in redox metabolism, porphyrin metabolism, and protein biosynthesis [43,44]. Elevated during the cell cycle of prophase, alanine, aspartate, and glutamate metabolism was first thought to be related to energy metabolism such as glycolysis and the TCA cycle [45,46,47,48]. Glutamate is converted to glutamine and α-ketoglutaric acid, which then enters the TCA cycle and participates in the production of ATP [45,49]. Aspartate is converted to alanine and then to pyruvate, which is further converted to acetyl-CoA under the action of branched-chain ketoacid dehydrogenase (BCKDH) to enter the TCA cycle [46,47,48]. Given the absence of mitochondria in human RBCs, the redox metabolism pathway is likely involved in rCPA-induced hemolysis. Using clues from metabonomic analysis, we explored the role of extracellular O_2_ and ROS in rCPA-induced hemolysis and found that ROS is involved in rCPA-induced hemolysis and is produced by NADPH oxidase. Meanwhile, the NADPH oxidase inhibitor we used also significantly inhibited rCPA-induced hemolysis, which also clarified that ROS played a role in this process. This is consistent with a previous study that showed that rabbit neutrophils treated with rCPA had a rapid but transient increase in ROS [50]. We verified the presence of elevated ROS in rCPA-induced hemolysis, and that the production of ROS increased with increasing hemolysis time. In addition, recent studies have shown that overactivation of the P2X_7_ receptor leads to a large release of ATP and excess production of ROS, and the selective P2X_7_ receptor inhibitor BBG reduces the production of ROS [31]. Therefore, we posit that the activation of P2 receptors is closely related to the production of ROS. Studies have shown that there are three ways to activate NADPH oxidase, including p38-MAPK, cx43, and the MEK/ERK pathway [51]. We further explored which pathway was involved in the activation of NADPH and found that the MEK1/ERK1 pathway is involved in the activation of NADPH oxidase and the progress of rCPA-induced hemolysis. We used inhibitors of several pathways to finally ascertain that cx43 and MEK1/ERK1 are the downstream pathway of P2 receptor activation by rCPA-induced hemolysis (Figure 8). ROS increase has also been reported in other toxin-induced hemolysis. Recent literature suggests that ETX-induced hemolysis is mediated by the metal-catalyzed oxidation of the swell-induced nucleotide-sensitive ICln chloride channel and the lysis of cells may rely on the production of ROS [52]. In vivo studies in mice also showed that inhibitors of NADPH oxidase could significantly reduce rCPA-induced ROS elevation, which adds to our physiological understanding of RCPA-induced hemolysis, not only in isolated RBCs. Taken together, we infer that ROS are a common cause of toxin-induced hemolysis.

In conclusion, we confirmed that rCPA-induced hemolysis in erythrocytes is related to the activation of P2 receptors, especially P2X_1_ and P2X_7_. Moreover, we found that the activation of P2 receptors led to the activation of NADPH oxidase through the MEK1/ERK1 and PI3K/Akt pathways and that the increased production of ROS eventually causes the lysis of erythrocytes. The mechanism by which rCPA activates P2 receptors and the relation between the activation of P2 receptors and the production of ROS were preliminarily revealed. In addition to this, it has been previously shown that CPA can directly bind to target cell membranes through the C-domain, leading to the toxin-dependent hydrolysis of phosphatidylcholine in liposomes [53]. Therefore, we speculate that this may also be one of the causes of red blood cell damage (Figure 8). We also demonstrate the presence of a common signaling pathway between pore-forming and non-pore-forming toxin-induced hemolysis, in which P2 receptors may play a key role, and that the production of ROS is most likely the main cause of hemolysis. These findings help to clarify the mechanism of action of rCPA and other hemolysins and also provide insight into potential candidate drugs for the treatment of rCPA-induced diseases.

However, in this study, we did not test whether cell membrane phospholipid hydrolysis plays a role in rCPA-induced hemolysis; therefore, future experimental validation of mutants with reduced cell surface lipid modification activity using CPA is warranted. In addition, the pathway of ATP production and ion changes during rCPA-induced hemolysis are still unclear, and further experiments are needed to verify whether the inhibitors of the hemolysis pathway we found are effective in the treatment of rCPA poisoning.

## 4. Materials and Methods

### 4.1. Materials

Pyridoxal phosphate-6-azo (benzene-2,4-disulfonic acid) tetrasodium salt hydrate (PPADS), Suramin, MRS2179, Brilliant Blue G(BBG), ATP-2′, 3′-dialdehyde (OxATP), SL327, carbenoxolone disodium salt, mefloquine, apyrase, BIRB 796, DPI (Diphenyleneiodonium), and BIX02189 were purchased from Sigma-Aldrich (St. Louis, MO, USA). NF023, A438079, KN-62, MRS2211, Evans Blue, tetrasodium salt, Perifosine, API-2, PD334581, TCS ERK 11e, FR 180204, and ERK5-IN-1 were purchased from Tocris Bioscience (Abingdon, UK). GKT136901, VAS2870, ML171, and Setanaxib were purchased from Med Chem Express (St. Madison, Green Bay, WI, USA). The ATP assay kit and fluorometric intracellular ROS kit were purchased from Sigma-Aldrich (St. Louis, MO, USA).

### 4.2. Preparation of Erythrocytes

Human blood was collected by venipuncture from healthy volunteers with evacuated blood collection tubes containing K_2_EDTA. After the mice were anesthetized with CO_2_, blood was collected from the mice in collection tubes containing K_2_EDTA. The erythrocytes were washed three times in 0.01 M PBS at centrifuged 1000× *g* at 4 °C for 10 min in a centrifuge (Eppendorf, Hamburg, Germany). The supernatant was removed and the isolated erythrocytes were re-suspended with PBS buffer to obtain a 5% red blood cell suspension and stored at 4 °C.

### 4.3. Preparation of Recombinant Toxin

Our laboratory constructed the recombinant plasmid pTIG-his-cpa that expressed rCPA. rCPA proteins were produced by *E. coli* BL21 (DE3) cells. Expression and purification of the toxin were conducted as previously described [14].

### 4.4. Measurements of the Hemolytic Activity

We prepared 5% erythrocyte solution suspensions by diluting red blood cells (RBCs) with PBS (containing 1 mM CaCl_2_). Purified rCPA (0~50.4 μM, 100 μL) was added to the 5% RBC suspension (the test group), PBS (the negative control group), and 2% TritonX-100 solution (the positive control group). To explore the effect of temperature on rCPA hemolysis, a series of concentrations of rCPA (0~12.6 μM) were added to RBCs and incubated at 4 °C, 25 °C, 37 °C, and 42 °C for 1h. And in subsequent experiments, all groups (the test group needed to be treated with or without antagonists first) were incubated up to 1 h in a constant-temperature mixer (Thermomixer comfort Eppendorf, Hamburg, Germany) with constant swirling (37 °C, 300 rpm), and hemolytic activity was determined by measuring the absorbance of the centrifuged (1000× *g*, 4 °C, 15 min) supernatants at 540 nm. The effect of osmotic pressure changes on hemolysis was verified using a range of concentrations of sucrose solution instead of the antagonist. The hemolytic assay was repeated three times for each group. Relative hemolysis was calculated as the ratio of the experimental group to the control group. The hemolysis value of the control group without an antagonist in each figure is generally defined as 1.

### 4.5. Scanning Electron Microscopy

We stopped the hemolysis reaction at 0, 5, and 30 min. Samples were first fixed overnight with pre-cooled PBS (pH 7.4) containing 2.5% glutaraldehyde (4 °C), washed with PBS, and dehydrated with acetone and series gradient alcohol. The samples were dried in a low vacuum, baked, and plated with gold. Finally, cells and untreated controls were analyzed and imaged using an S-3400N scanning electron microscope (HITACHI, Tokyo, Japan).

### 4.6. Measurement of Cellular ATP

Cellular ATP content was determined by the luminescence method using an ATP assay kit according to the manufacturer’s protocol. rCPA was incubated with RBCs for 0, 5, 10, 30, 60, and 90 min at 37 °C and 300 rpm. The supernatant was collected after centrifugation (1000× *g*, 4 °C, 10 min). The supernatant (50 μL) was mixed with nucleotide-releasing buffer (50 μL) and ATP-monitoring enzyme (1 μL), and then the mixture was incubated at room temperature for 30 min away from light. Chemiluminescence at 570 nm was measured using a Varioskan Flash Multimode Reader (Thermo Fisher Scientific, Waltham, MA, USA) and the software SkanIt 7.0.

### 4.7. Measurement of Intracellular ROS

The intracellular ROS was determined using a fluorometric intracellular ROS kit following the manufacturer’s protocol. The master reaction mix was added (100 µL per well) into the reaction system at different times, and then the erythrocytes were incubated at 37 °C for 1 h. Fluorescence intensity at λ_ex_ = 490/λ_em_ = 525 nm was measured. The sample was read using a Varioskan Flash Multimode Reader (Thermo Fisher Scientific, Waltham, MA, USA).

### 4.8. In Vivo Experiments in Mice

Mice were treated with DPI (2 mg/kg/d) and NOX inhibitor mixture (20 mg/kg/d) for 3 consecutive days in advance, after which rCPA (590 μg/kg) was injected intraperitoneally. The PBS injection group was used as a negative control, and the rCPA (590 μg/kg) injection group was used as a positive control. One hour later, the mice were anesthetized with CO_2_, and blood samples were drawn to detect ROS levels in plasma.

### 4.9. Metabonomic Analysis

Normal RBCs and CPA-treated RBCs were put into liquid nitrogen to terminate the reaction. Samples were then centrifuged at 12,000 ×g after ultrasound, the supernatant was mixed with twice the volume of phosphate buffer (0.2 M PBS, 80% H_2_O, 20% D_2_O, pH = 7.4) to eliminate the effect of pH on chemical shift, and an appropriate amount of Dss (2, 2-Dimethyl-2-silapentane-5-sulfonic acid) added as the zero of the chemical shift. After preparation, samples were sent to Metabo-Profile for high-performance liquid chromatography–mass spectrometry (HPLC-MS) analysis and nuclear magnetic resonance (NMR) analysis. The original data files generated by UPLC-MS/MS were peak-integrated, calibrated, and quantified by MassLynx software (v. 4.1, Waters, Milford, MA, USA). iMAP software (v. 1.0, Metabo-Profile, Shanghai, China) was used for statistical analysis, including principal component analysis (PCA), orthogonal partial least squares Discrimination Analysis (OPLS-DA), univariate analysis, and pathway analysis.

### 4.10. Statistical Analysis

Values are the mean ± SD (*n* = 3) of at least three independent biological replicates for each experiment. The differences between the data were tested by an independent sample *t* test, and *p* < 0.05 was considered statistically significant. Data analysis was completed in GraphPadPrism9.0 software.

## Figures and Tables

**Table 1:** Antagonists tested.

Antagonists	Target	Human Erythrocytes	Murine Erythrocytes
PPADS	P2 receptors	√	√
Suramin	P2 receptors	√	√
Evans Blue	P2X	√	√
NF023	P2X_1_	×	√
BBG	P2X_1_/P2X_7_	√	√
OxATP	P2X_1_/P2X_7_	√	√
A438079	P2X_7_	×	×
KN-62	P2X_1_/P2X_7_	×	×
Mg^2+^	P2X_7_	√	×
MRS2179	P2Y_1_	×	×
MRS2211	P2Y_13_	√	√
Carbenoxolone	Pannexin 1	×	×
Mefloquine	Pannexin 1	×	×

Note: “√” indicates that the inhibition can inhibit the hemolysis caused by CPA; “×” indicates that the inhibitor failed to inhibit hemolysis caused by CPA.

**Table 2:** Inhibitors of different subtypes of NOX.

Inhibitors	Target NOX Subtype				
	NOX1	NOX2	NOX3	NOX4	NOX5
GKT136901	√	√		√	√
Setanaxib	√	√		√	√
VAS2870		√			
ML171	√	√	√	√	

Note: “√” indicates that the inhibitor can inhibit the activity of the NOX subtype.

## Data Availability

The datasets generated during and/or analyzed during the current study are available from the corresponding author on reasonable request.

## Supplementary Materials

The following supporting information can be downloaded at: https://www.mdpi.com/article/10.3390/pathogens13060454/s1, Figure S1. SDS-PAGE and Western blot of rCPA after purification. Figure S2. SDS-PAGE of Hlb after purification. Figure S3. Influence of different concentrations of PPADS on Hlb-induced hemolysis of human and murine erythrocytes. Figure S4. Mg^2+^ did not alter the enzymatic activity of CPA. Figure S5. The effect of ADP on rCPA-induced hemolysis.
