# Supplementary material for: Purinergic Receptor Antagonists Inhibit Hemolysis Induced by Clostridium perfringens Alpha Toxin
Source: Pathogens. 2024 May 27;13(6):454. doi: 10.3390/pathogens13060454 (PMC11206506; doi:10.3390/pathogens13060454)
Supplement: Supplementary file 1 [file pathogens-13-00454-s001.zip › pathogens-2933375-supplementary.pdf]

Supplementary information

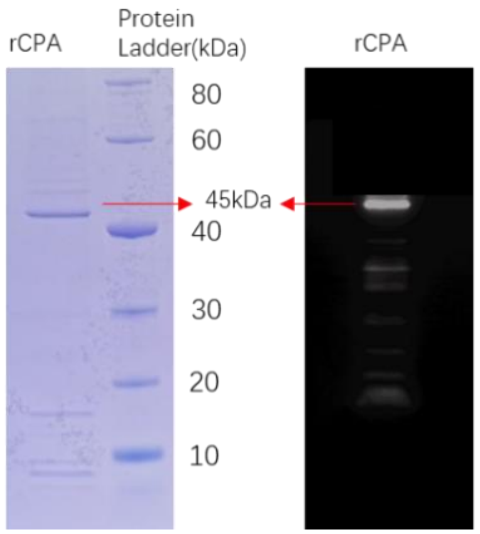

Figure S1. SDS-PAGE and Western blot of rCPA after purification.

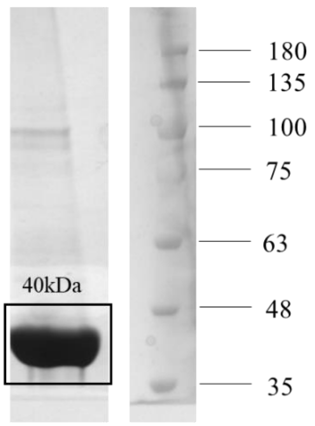

Figure S2. SDS-PAGE of Hlb after purification.

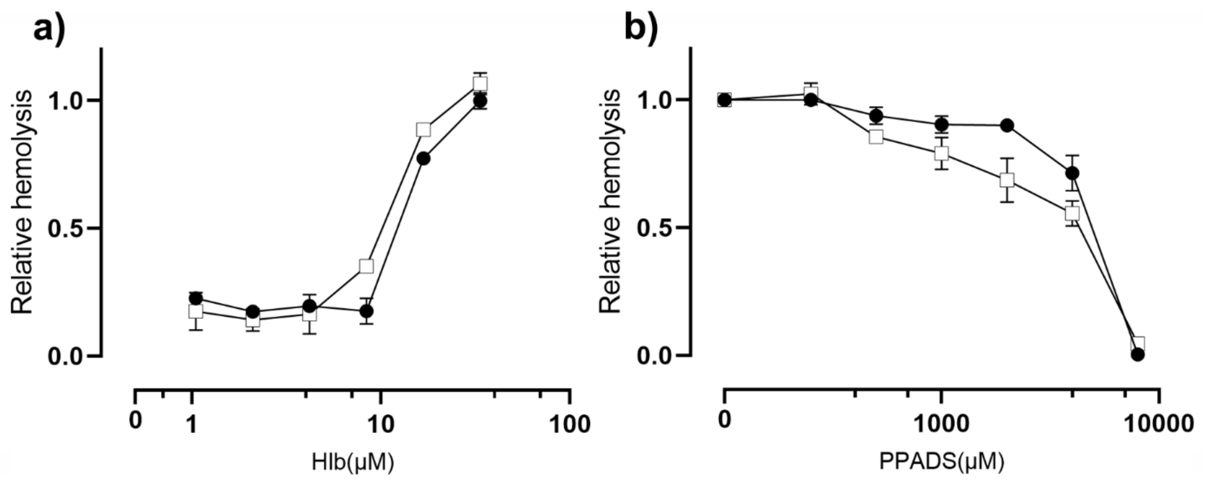

Figure S3. Influence of different concentrations of PPADS on Hlb-induced

hemolysis of human and murine erythrocytes. (a) Human and murine erythrocytes were treated with different concentrations of Hlb (0 - 33.5 μM). (b) PPADS (250 μM-8 mM) inhibits Hlb-induced hemolysis of human and murine erythrocytes.

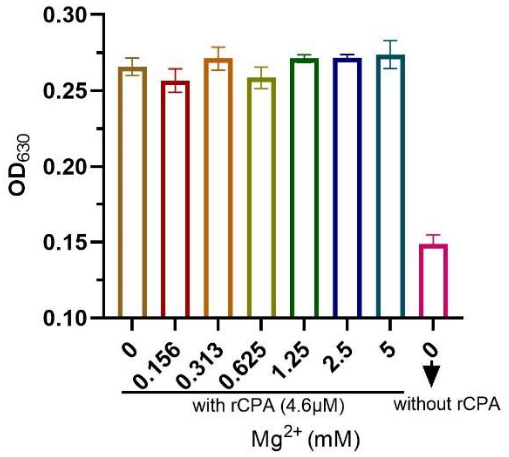

Figure S4. Mg^{2+} did not alter the enzymatic activity of CPA. The enzymatic activity of CPA was proportional to the turbidity of the solution, with higher OD_{630} values indicating higher enzymatic activity of rCPA. The enzymatic activity of CPA was not decreased by increasing Mg^{2+} concentration.

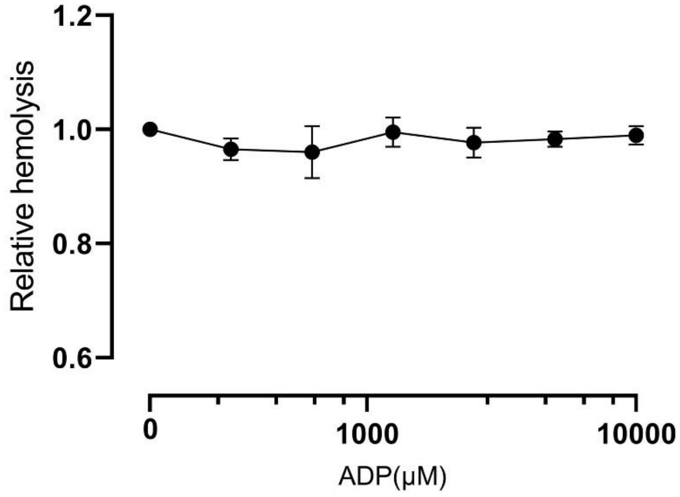

Figure S5. The effect of ADP on CPA-induced hemolysis. P2Y13 receptor

agonist ADP does not inhibite CPA-induced hemolysis.
